# Examining temporal features of BOLD-based cerebrovascular reactivity in clinical populations

**DOI:** 10.3389/fneur.2023.1199805

**Published:** 2023-06-15

**Authors:** Kayley-Jasmin Marchena-Romero, Xiang Ji, Rosa Sommer, Andrew Centen, Joel Ramirez, Joshua M. Poulin, David Mikulis, Michael Thrippleton, Joanna Wardlaw, Andrew Lim, Sandra E. Black, Bradley J. MacIntosh

**Affiliations:** ^1^Department of Medical Biophysics, University of Toronto, Toronto, ON, Canada; ^2^Hurvitz Brain Sciences Program, Sunnybrook Research Institute, Toronto, ON, Canada; ^3^Dr. Sandra Black Centre for Brain Resilience and Recovery, Toronto, ON, Canada; ^4^Institute of Medical Sciences, University of Toronto, Toronto, ON, Canada; ^5^Division of Neuroradiology, Joint Department of Medical Imaging, University Health Network, Toronto, ON, Canada; ^6^Department of Medical Imaging, University of Toronto, Toronto, ON, Canada; ^7^Brain Research Imaging Centre, Centre for Clinical Brain Sciences, UK Dementia Research Institute Centre, The University of Edinburgh, Edinburgh, United Kingdom

**Keywords:** cerebrovascular reactivity, inter-regional heterogeneity, non-parametric, sleep apnea, small vessel disease, blood oxygenation level dependent functional magnetic resonance imaging

## Abstract

**Background:**

Conventional cerebrovascular reactivity (CVR) estimation has demonstrated that many brain diseases and/or conditions are associated with altered CVR. Despite the clinical potential of CVR, characterization of temporal features of a CVR challenge remains uncommon. This work is motivated by the need to develop CVR parameters that characterize individual temporal features of a CVR challenge.

**Methods:**

Data were collected from 54 adults and recruited based on these criteria: (1) Alzheimer’s disease diagnosis or subcortical Vascular Cognitive Impairment, (2) sleep apnea, and (3) subjective cognitive impairment concerns. We investigated signal changes in blood oxygenation level dependent (BOLD) contrast images with respect to hypercapnic and normocapnic CVR transition periods during a gas manipulation paradigm. We developed a model-free, non-parametric CVR metric after considering a range of responses through simulations to characterize BOLD signal changes that occur when transitioning from normocapnia to hypercapnia. The non-parametric CVR measure was used to examine regional differences across the insula, hippocampus, thalamus, and centrum semiovale. We also examined the BOLD signal transition from hypercapnia back to normocapnia.

**Results:**

We found a linear association between isolated temporal features of successive CO_2_ challenges. Our study concluded that the transition rate from hypercapnia to normocapnia was significantly associated with the second CVR response across all regions of interest (*p* < 0.001), and this association was highest in the hippocampus (*R*^2^ = 0.57, *p* < 0.0125).

**Conclusion:**

This study demonstrates that it is feasible to examine individual responses associated with normocapnic and hypercapnic transition periods of a BOLD-based CVR experiment. Studying these features can provide insight on between-subject differences in CVR.

## 1. Introduction

The cerebrovascular network is tightly regulated to maintain appropriate cerebral blood flow in the presence of systemic changes in blood pressure (1). Changes in blood pressure, neuronal activity, or arterial blood gases can elicit strong cerebrovascular responses and changes in vascular tone. Increasing the fraction of inhaled CO_2_ is a common cerebrovascular reactivity (CVR) challenge, as it increases the partial pressure of CO_2_ in arterial blood. There are two distinct transition phases that occur during a CVR challenge. The transition from a normocapnic to hypercapnic state is facilitated by vasodilation. During hypercapnia there is a decrease in intravascular pH, causing a cascade of chemical reactions in the endothelial and smooth muscle components of the vessel wall that increase the vessel lumen diameter, resulting in an influx of oxygenated blood. Upon cessation of the hypercapnic stimulus (i.e., returning to room air), a reversal effect occurs where blood vessels transition back to a physiological baseline. This process is induced by vasoconstriction, a physiological component of the hypercapnic response, whereby a passive decrease in arterial CO_2_ occurs once the hypercapnic stimulus ends. A resultant CO_2_ washout is promoted through a negative feedback loop where a decrease in local CO_2_ results in a smaller vessel diameter (2). These dynamic structural and physiological responses to CO_2_ influx and efflux can be influenced by cerebrovascular properties such as tissue type and vascular resistance (3, 4).

CVR can be assessed through dynamic functional neuroimaging to indirectly measure the vascular response to a vasoactive stimulus, providing a means to visualize and quantify cerebrovascular function *in-vivo* (2). Metrics of CVR have been demonstrated to have utility in the characterization of conditions as diverse as stroke (5, 6), dementia (7–9), small vessel disease (10, 11), obstructive sleep apnea (12–14), and Moyamoya disease (15, 16). Dynamic responses to a CVR challenge can be imaged using blood oxygenation level dependent (BOLD) Magnetic Resonance Imaging (17–19) as well as other nuclear medicine approaches (11, 20). The contrast for BOLD-MRI images arises from changes in the local concentration of oxygenated to deoxygenated blood. What drives this shift in concentration is dependent on multiple characteristics of brain physiology, such as cerebral blood flow and cerebral blood volume. BOLD-MRI indirectly measures the longitudinal changes that occur when these parameters are manipulated by a hypercapnia challenge. This non-invasive imaging technique offers a high contrast-to-noise ratio and an adequate temporal resolution (3, 19, 21–23).

CVR is typically calculated using a general linear model (GLM) that relies on model parameters to yield a single CVR estimate. For example, the end-tidal CO_2_ (PETCO_2_) trace is typically convolved with a hemodynamic response function (HRF) in a GLM-CVR analysis. Other modeling approaches account for systematic differences between the PETCO_2_ and BOLD signal; these examples include a hemodynamic lag term, refined HRF, transfer functions, and non-linear analyses (24–26). However, linear methods often assume that the response pattern of the BOLD and PETCO_2_ signal are highly consistent. Diseases that affect the cerebrovasculature may challenge this assumption. Furthermore, the conventional CVR measure summarizes the relative change in BOLD signal for multiple challenges in succession despite potential information that could be gained from assessing each challenge individually. Given that cerebrovascular variability may reflect pathophysiology and CBF differences, we look to examine the more granular nuances of the BOLD signal response to CO_2_ challenges in succession (27, 28).

The primary objective of this study is to develop a non-parametric CVR metric that describes the change in BOLD signal induced by the dilatory response to hypercapnia, while circumventing the need for model-fitting, and allowing the quantification of individual CVR responses. The Theil-Sen Estimator (Sen’s Slope) is a non-parametric regression model and can be used as a CVR metric to characterize the relative change in magnitude of the BOLD signal induced by individual dilatory responses to hypercapnia. Our second objective is to characterize the BOLD signal decrease that occurs during the transition period following a hypercapnia challenge. We hypothesize that the rate of BOLD signal decline during a transition period from hypercapnia to normocapnia will be significantly associated with the non-parametric CVR measure of the following hypercapnia challenge. These metrics are extracted in two clinical populations: individuals with sleep apnea and cognitive concerns. Regional differences in response to hypercapnia are of interest in these clinical populations, hence we conduct a region-of-interest (ROI) analysis. The hippocampus is part of the physiological brain alterations seen in cognitive impairment and/or sleep apnea (29–32). Second, the insula is highly organized in its regulation of the autonomic nervous system, which includes cardiovascular responses to stimuli (33). Third, the thalamus is highly implicated in both sleep disorders and cognitive impairment (34, 35). Fourth, the centrum semiovale is relevant to CVR as it has lower cerebral blood flow compared to grey matter, it has limited collateral arterial blood supply, and is proximal to the draining medullary veins that are implicated in vascular brain dysfunctions (36–38).

## 2. Methods

### 2.1. Participants

Participants were recruited at Sunnybrook Research Institute as part of an on-going international study (10) and were either: (1) adults with Alzheimer’s Disease, Mild Cognitive Impairment, or subjective memory concerns seen at a cognitive neurology clinic, or (2) adults with sleep apnea seen at a sleep neurology clinic. A third retrospective cohort comprised individuals with memory complaints (39). All participants gave written informed consent. This research was approved by Sunnybrook Health Sciences Centre Research Ethics Board.

Group 1: Participants underwent clinical testing to ascertain Alzheimer’s disease diagnosis as per the National Institute on Ageing-Alzheimer’s Association (NIA-AA) (40), Mild Cognitive Impairment as per the Albert diagnostic guidelines (41), subcortical Vascular Cognitive Impairment, or subjective complaints. Group 2: Participants were newly diagnosed with sleep apnea based on an apnea hypopnea index ≥15 and at an oxygen desaturation index of 10 de-saturations per hour on diagnostic polysomnography, accompanied by subjective sleepiness, and have never been treated for sleep apnea. Exclusion criteria included: history of major stroke or other central nervous system diseases, use of alpha-blockers, persistent non-sinus arrhythmia, severe pulmonary or cardiac diseases including chronic obstructive pulmonary disease and congestive heart failure, waking saturation of <90%, and history of panic disorder. Group 3: Participants from a retrospective data collection at Sunnybrook Health Sciences Centre (42) were accessed and comprised of older adults with subjective memory complaints; age and sex were matched to Group 1.

For all three groups, participants were excluded based on: contraindications to MRI safety, history of significant head trauma, brain tumours, or hydrocephalus, significant drug or alcohol abuse, and current unstable cardiac disease.

### 2.2. MRI data collection

MRI data for Group 1 and Group 2 were collected using a 3 Tesla (3 T) Siemens Prisma system scanner (Siemens Medical Solutions, Erlangen, Germany) with a 32-channel head coil reception and body coil transmission. T1-weighted images were collected for registration with functional images and to define structural regions of interests to be segmented. Functional (i.e., BOLD-fMRI) images were collected using a simultaneous multi-slice echo planar imaging (SMS-EPI) pulse sequence (43). T2-weighted FLAIR sequence images were collected for white matter hyperintensity (WMH) quantification.

MRI data for Group 3 were collected using a 3 T Philips Achieva system scanner (Philips Healthcare, Best, Netherlands). T1-weighted and T2-weighted FLAIR images were collected on this scanner. BOLD-fMRI data were collected using a single-shot EPI. MRI parameters are summarized in Table 1.

**Table 1:** Overview of imaging parameters for each group.

	Group 1	Group 2	Group 3
T1-weighted acquisition			
Repetition time (TR), ms	2,500	2,500	9.5
Flip angle, degrees	7	7	8
Gap, mm	0.5	0.5	-
Echo time (TE), ms	4.37	4.37	2.3
Matrix size	256 × 256 × 192	256 × 256 × 192	384 × 384 × 140
In-plane/spatial resolution, mm^3^	1	1	1.2
Slices, number	192	192	140
Slice thickness, mm	1	1	1.2
BOLD acquisition	multi-shot EPI	multi-shot EPI	single-shot EPI
Repetition time (TR), ms	1,550	1,550	2000
Flip angle, degrees	67	67	90
Gap, mm	0	0	0
Echo time (TE), ms	30	30	30
Acceleration Factor	2	2	-
Voxel size, mm	2.5 × 2.5 × 2.5	2.5 × 2.5 × 2.5	3.6 × 2.9 × 3
Slices, number	50	50	40
Slice thickness, mm	2.5	2.5	3
Volume number	478	478	255
T2-weighted acquisition	FLAIR	FLAIR	FLAIR
Repetition time (TR), ms	5,000	5,000	9,000
Flip angle	T2 var	T2 var	-
Inversion time (TI), ms	1800	1800	2,800
Echo time (TE), ms	388	388	125
Matrix size	256 × 256 × 192	256 × 256 × 192	240 × 217
In-plane/spatial resolution, mm^3^	1	1	3.3
Slices, number	192	192	52
Slice thickness, mm	1	1	3
Field of view (mm)	256	256	240

### 2.3. Hypercapnia challenge

The CVR set-up used for Group 1 and Group 2, Experimental Design A, was established as part of a collaborative two-site study (10). An enriched-CO_2_ gas blend contained in a gas cylinder was fed *via* plastic tubing through the MRI waveguide to the tight-fitting face mask worn by the participant in the MRI. Gas administration was interleaved with room air “baseline” periods to produce a boxcar breathing paradigm. The challenge was administered as follows: 2 min of room air, 3 min of 6% CO_2_ air, 2 min of room air, 3 min of 6% CO_2_ air, and 2 min of room air. CO_2_ and O_2_ gas traces were monitored using two gas analyzers, O2100C and CO2100C, that are part of the BIOPAC® respiratory gas analysis modules (BIOPAC Systems, California, United States). The hypercapnic challenge for Group 3, Experimental Design B, was administered using a RespirAct Gen3 breathing circuit (Thornhill Research, Toronto, Canada) to reach a targeted 10 mmHg PETCO_2_ change. Unlike the first breathing paradigm, hypercapnic gas was administered in unequal timing blocks: 45 s rest, 45 s on, 90 s rest, followed by a second dilation challenge of 120 s and a final rest of 180 s. This external data source was retroactively analyzed, and differences in the hypercapnic challenge are not intentional.

### 2.4. BOLD data processing

BOLD data were pre-processed using parameters appropriate for the image size. Data were motion-corrected using FSL MCFLIRT (FSL, version 5.0, http://fsl.fmrib.ox.ac.uk), spatially smoothed with a FWHM Gaussian Kernel (Group 1 and 2: 3 mm, Group 3: 5 mm), a high-pass filter was applied [Group 1 and 2: 3.18 × 10^−4^ Hz (500 s), Group 3: 1.60 × 10^−3^ Hz (300 s)] to remove drift in the signal, and slice-timing correction was performed. An initial set of BOLD volumes were discarded to account for signal equilibrium (Group 1 and 2: 25 volumes, Group 3: 5 volumes).

### 2.5. Region-of-interest generation

**Figure 1:**
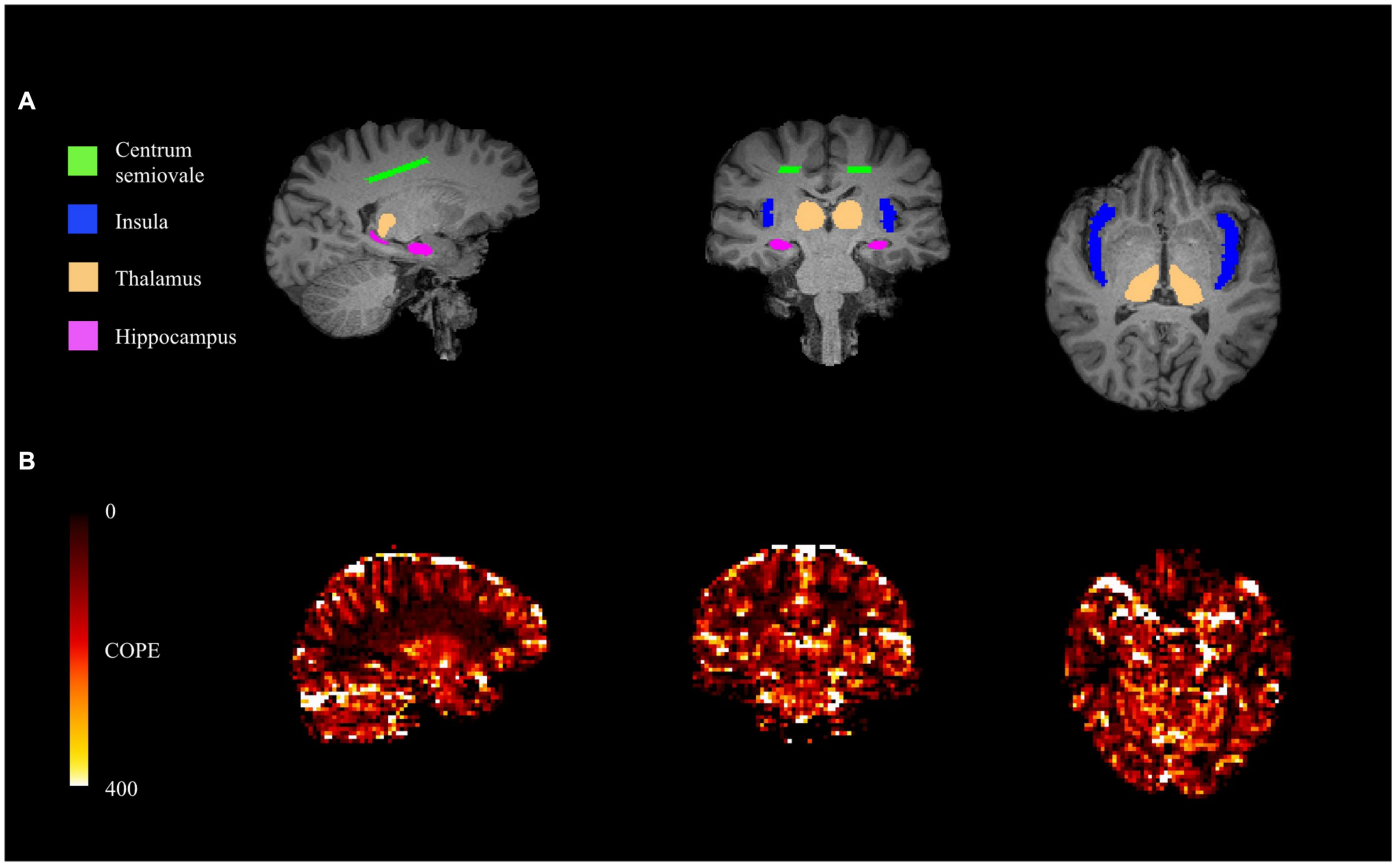
From left to right: sagittal (posterior to anterior), coronal (right to left), and axial (right to left) views of T1-space ROIs **(A)**. From left to right: sagittal (posterior to anterior), coronal (right to left), and axial (right to left) views of COPE-CVR map **(B)**.

Four pre-defined bilateral ROIs were identified for hypothesis testing in this study, which included the insula, thalamus, hippocampus, and centrum semiovale. The insula was segmented using a MNI152 standard space template and MNI structural atlas (44, 45). The hippocampus was segmented in native T1-space using the convolutional neural network-based tool HippMapp3r (hippmapp3r.readthedocs.io) (46). The thalamus was segmented using FIRST in FSL using the native T1 image. Finally, the bilateral centrum semiovale was defined as contiguous voxels of white matter in an MNI152 standard space axial plane that is 3 mm superior to the top of the lateral ventricles. The standard space masks were transformed into native T1-space. Bilateral ROIs in T1-space are conceptualized (Figure 1A). For each of the ROIs, the masks were down-sampled from T1-space to BOLD-space to generate BOLD-CVR time series data. All registrations were performed using FSL FLIRT (FSL, version 5.0, http://fsl.fmrib.ox.ac.uk).

### 2.6. Isolation of transition phases during CVR challenge

Physiologically distinct periods were isolated according to the experimental breathing paradigm of the group (refer to Section 2.3) and are summarized in Table 2. The first and second non-parametric CVR metrics (CVR-1 and CVR-2) arise from the transition periods that describe the BOLD signal response to the first and second administration of CO_2_-rich air, respectively. The hypercapnia to normocapnia transition period describes the BOLD response as it returns to room air condition, specifically the first half of the rest period. These analytical time windows were applied consistently for all CVR data.

**Table 2:** Analytical time windows.

	Time windows (in s)			
Experimental design	CVR-1	CVR-2	Hyper-to-normocapnia transition period 1	Hyper-to-normocapnia transition period 2
Experimental Design A	40 s–280 s	360 s–580 s	280 s–360 s	580 s–660 s
Experimental Design B	10 s–80 s	135 s–280 s	80 s–135 s	280 s–390 s

### 2.7. Transition from normocapnia to hypercapnia

We used Sen’s Slope as a non-parametric metric to quantify the percent change in BOLD signal when transitioning from normo-to-hypercapnia conditions; this approach circumvents the need to fit the BOLD signal to the PETCO_2_ signal and is robust to outlier measurements that may occur due to imaging noise. CVR-1 describes the response to the first CO_2_ challenge, while CVR-2 describes the response to the second challenge. BOLD signal differences were calculated between every pair of values in the time series excerpt, resulting in a total of *n*(*n*−1)/2 pairs of data. These values were then divided by the interval range corresponding to the calculated signal difference. The Sen’s Slope was based on the median rate of BOLD change from among each of the calculated values (Equation 1), followed by multiplying by the number of data points within the dilation period, dividing the mean baseline BOLD signal prior to the hypercapnia challenge, and yielding a unit-less BOLD percent signal change (Equation 2). BOLD percent signal change is then converted to a CVR metric by dividing by the change in PETCO_2_ (Equation 3).


(1) $$\mathrm{Se}n^{\prime }s\;\mathrm{Slope}=\mathrm{median}\left(\frac{x_{j}-x_{i}}{j-i}\right)$$

Where *x_i_* and *x_j_* are BOLD signal values at times *i* and *j* (*j* > *i*).


(2) $$\%\Delta \mathrm{BOLD signal}=\frac{\mathrm{Se}n^{\prime }s\;\mathrm{Slope}*\#\mathrm{data points}}{\mathrm{mean baseline BOLD signal}}*100$$

(3) $$\mathrm{CVR}=\frac{\%\Delta \mathrm{BOLD signal}}{\Delta {\mathrm{PETCO}}_{2}}$$

To compare this approach against a conventional method, we calculated the coefficient of parameter estimate (COPE) values based on the FSL feat tool.^1^ This CVR estimator uses the voxel with the highest agreement (i.e., COPE value) between the signal and experimental paradigm to produce a CVR estimate (Figure 1B). The COPE-CVR estimate is representative of the entire hypercapnia challenge, rather than individual responses.

### 2.8. Transition from hypercapnia to normocapnia

The BOLD signal will tend to decrease after each hypercapnia challenge. This signal recovery is related to vasoconstriction and the transition back to normocapnia. We approximate this response as the linear rate of change in a regression model (lm function in R, http://www.R-project.org) where the BOLD signal is modeled as a function of time starting from the transition period (Equation 4). The resultant parameter estimate (𝜏) was further normalized by the change in PETCO_2_ to account for the influence of CO_2_ on the BOLD signal (Equation 5).


(4) BOLDsignal=τ∗time+intercept

(5) $$\mathrm{Transition Rate}=\frac{\tau }{\Delta PET{\mathrm{CO}}_{2}}$$

### 2.9. Simulation

We evaluated non-parametric and parametric CVR estimates through simulated hypercapnia events. The BOLD signal response to CO_2_ inhalation can vary depending on several factors; to simulate this we considered two (fast and slow) responses. A fast response was approximated by a sigmoidal curve for the BOLD signal change, while a slow response was simulated as a slower ramp up. Increments of simulated noise were added to each response to produce a total of 4,000 normocapnia-to-hypercapnia simulations. To characterize temporal noise characteristics, a temporal signal-to-noise (tSNR) ratio was calculated by dividing the mean baseline signal by its standard deviation (47). The tSNR was used to evaluate the influence that incremental increases of white noise added to the simulated time series would have on the CVR estimates. Both non-parametric (Sen’s) and parametric (linear regression) slopes for the simulated BOLD signal change were determined. The regressor in the parametric approach was a convolution of the gamma-HRF and the experimental paradigm. An Akaike Information Criterion (AIC) estimate was computed for both models to compare which model had the most likelihood of producing simulated scenarios.

### 2.10. Statistical analysis

All statistics were performed in R (version 1.2.1335). We tested our hypothesis on the empirical data by conducting two regression models for each ROI. In the first regression model (CVR Model-1), we assess the effect of the transition rate on the non-parametric CVR estimate of the second hypercapnia challenge (CVR-2), while adjusting for age, sex, clinical population, and CVR experimental design differences. For the ‘clinical population’ confounder, Group 1 and Group 3 were pooled because they are a similar clinical population (e.g., cognitive concerns), while Group 2 participants were grouped separately. For the ‘experimental design’ confounder, Group 1 and Group 2 were pooled together due to similarities in experimental design. In the second regression model (CVR Model-2) we account for similarities between hypercapnia challenges in succession by orthogonalizing the transition rate relative to CVR-2 and considering the first CVR estimate as an additional explanatory variable. A *p*-value was considered significant if it reached *p* ≤ 0.0125, to account for 4 ROIs. Standardized parameter estimates and adjusted-*R*^2^ values are reported for each predictor. Pearson correlation estimates between transition rate, CVR-1, CVR-2, and COPE-CVR are reported for the thalamus to demonstrate the utility of characterizing individual temporal features of a CVR challenge.

## 3. Results

### 3.1. Simulation

**Figure 2:**
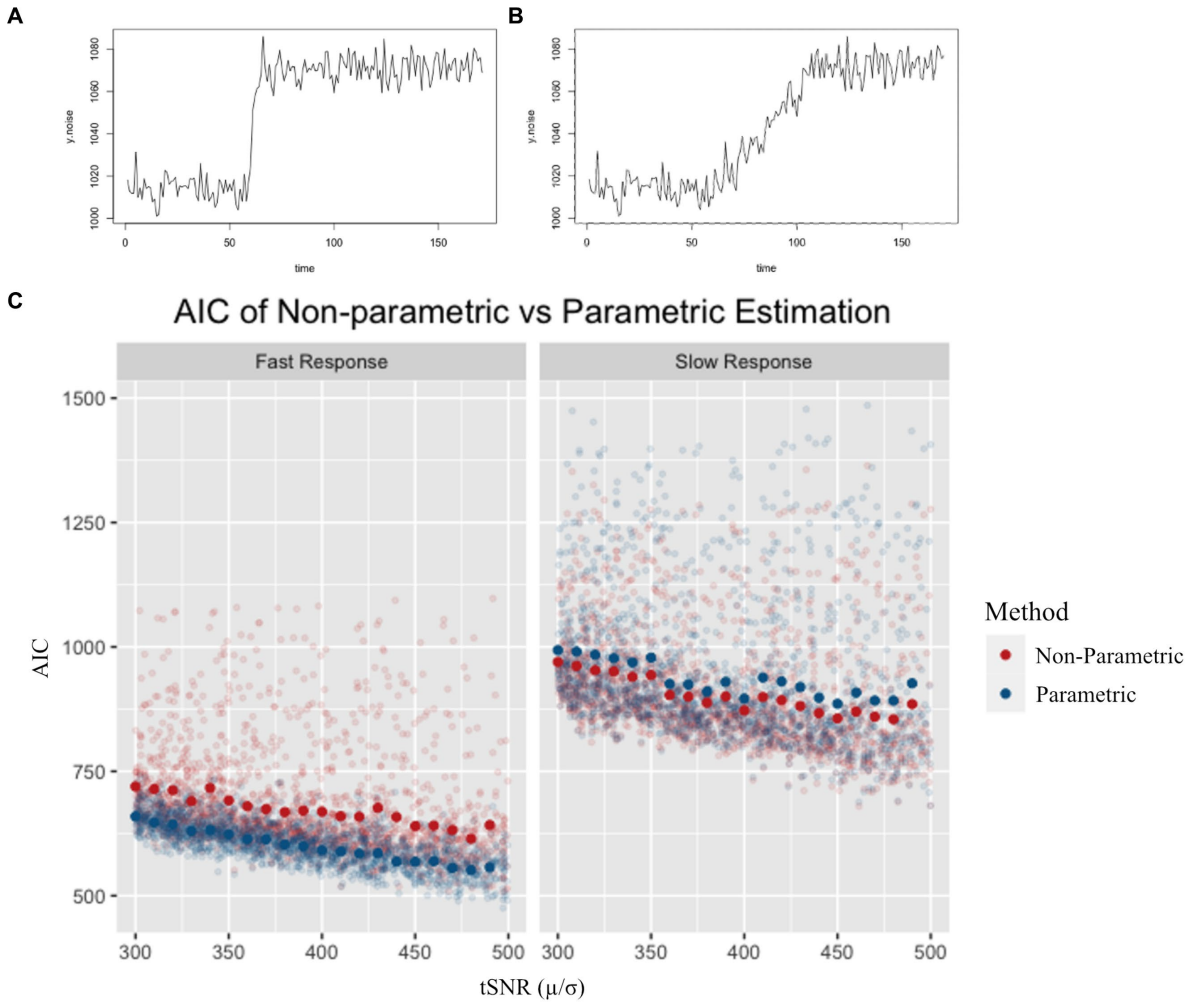
Example of a simulated fast **(A)** and simulated slow **(B)** response. AIC values for non-parametric and parametric models **(C)** are shown for a range of tSNR levels. Semi-transparent points represent individual simulation AIC results, while solid points represent the average AIC value for simulations across tSNR values. AIC was lower for the parametric model in the fast response simulation, and lower for the non-parametric model in the slow response simulation.

Simulation results revealed a marginal benefit of using the parametric method for fast CVR responses (Figure 2A), as reflected by lower AIC values and less variability compared to the non-parametric values. For the simulated slow response (Figure 2B), however, the non-parametric method had a marginally lower AIC than the parametric model. As expected, the fast responses had lower AIC values compared to the slow response (Figure 2C). There was a general trend of lower AIC as the tSNR increased for all simulations.

### 3.2. Empirical CVR

Cohort characteristics for the 54 participants in this study are provided in Table 3. Age, sex, years of education, Montreal Cognitive Assessment (MoCA) scores, and white matter hyperintensity (WMH) volumes are reported.

**Table 3:** Participant demographics.

	Group 1 (Alzheimer’s, MCI, subjective)	Group 2 (sleep apnea)	Group 3 (subjective cognitive concerns)
Group characteristics			
Sample (*n*)	20	20	14
Mean age ± SD (y)	70 ± 9	59 ± 12	72 ± 11
Sex (male/female)	9/11	13/7	3/11
Education (years)	16.0 [14.0, 18.0]	16.0 [15.5, 21.0]	16.5 [15.3, 19.0]
MoCA	23.5 [17.8, 27.2]	26 [23.2, 27.0]	25 [22.0, 29.0]
WMH volume (mm^3^)	5,770 [2,870, 28,500]	1,220 [486, 2,200]	36,100 [10,600, 56,600]

MOCA, Montreal cognitive assessment; WMH, white matter hyperintensity; SD, standard deviation. Values are presented as median [1st quartile, 3rd quartile].

**Figure 3:**
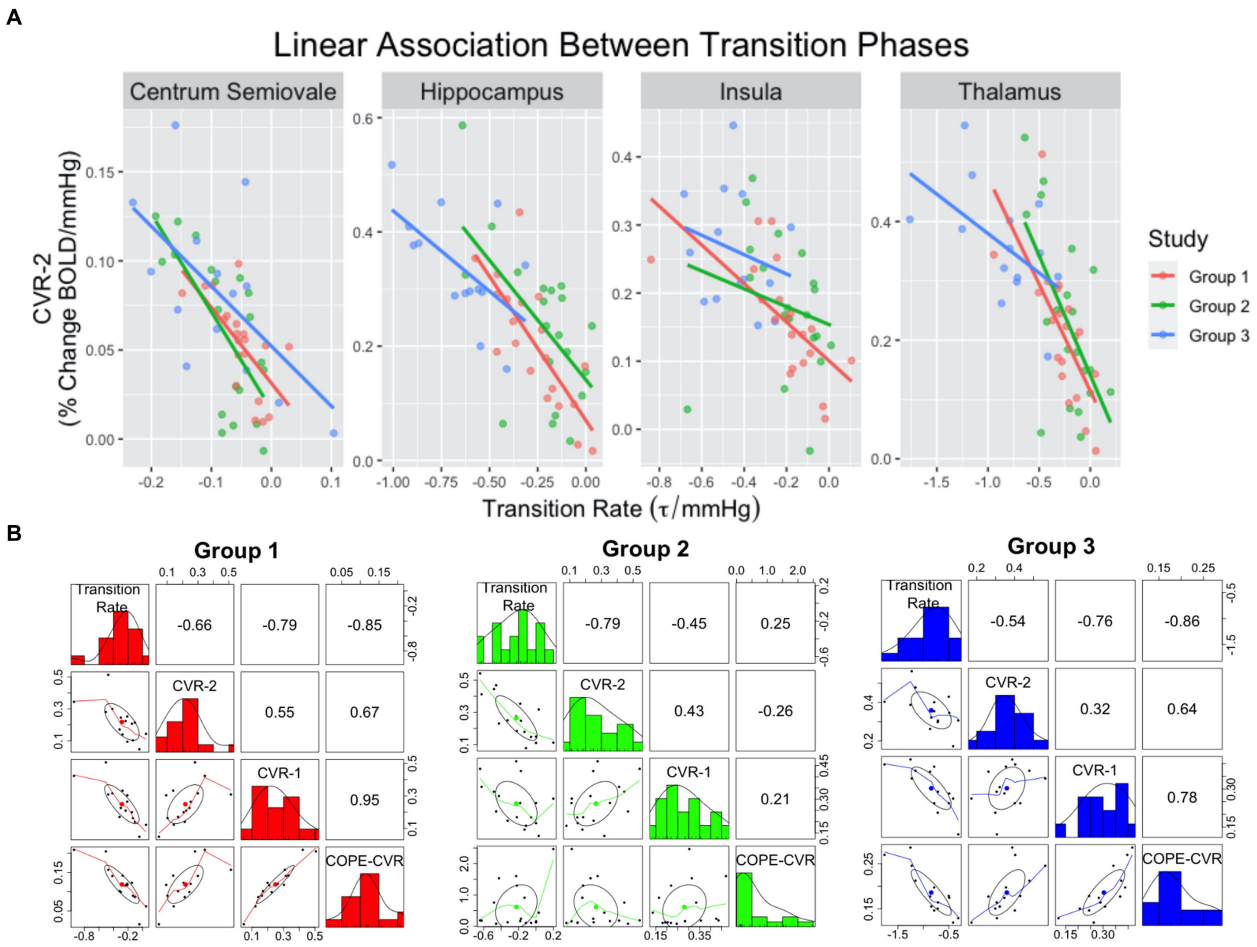
Scatterplots **(A)** demonstrating linear relationships between transition rate and CVR-2. The colored lines correspond to group assignment. Correlation plots **(B)** between transition rate, CVR-1, CVR-2, and COPE-CVR in the thalamus. For the correlation plots, the top-right values represent the *R*^2^ value between two variables, the middle-diagonal row is a histogram of values for a given variable, and the left-bottom plots represent scatterplots between two variables.

For CVR Model-1, the rate of BOLD signal decrease during the transition to normocapnia was significantly associated with the relative change in BOLD signal during a following hypercapnia event for each ROI, after accounting for age, sex, experimental design, and group as covariates (*p* < 0.008 for all ROIs) (Figure 3A). The strength of association was highest for the hippocampus (*R*^2^ = 0.51, *p* < 0.001), while the insula had the weakest association (*R*^2^ = 0.30, *p* > 0.0125) as reflected by the adjusted *R*-squared in Table 4. We observed that no other model confounders were significantly associated with the 2nd CVR metric in any ROI (*p* > 0.0125). In CVR Model-2, the two CVR metrics describing the first and second challenge were significantly associated with each other across all regions (*p* < 0.001) as expected due to the homogeneity between challenges. However, the adjusted transition rate was also significant (*p* < 0.008) for the thalamus, hippocampus, and centrum semiovale, but not for the insula (*p* = 0.38). Additionally, the CVR experimental design covariate was associated with dilation for the insula (*p* = 0.009), but no other covariates were significantly associated for the ROIs (*p* > 0.0125).

**Table 4:** CVR model results.

ROI	CVR Model-1		CVR Model-2		
	Transition rate (PE)	*R* ^2^	CVR-1 (PE)	Adjusted transition rate (PE)	*R* ^2^
Insula	−0.40*	0.30	0.48*	−0.12	0.40
Thalamus	−0.73*	0.43	0.57*	−0.42*	0.46
Hippocampus	−0.83*	0.51	0.52*	−0.58*	0.57
Centrum semiovale	−0.65*	0.43	0.49*	−0.46*	0.45

Standardized parameter estimates (PE) and adjusted *r*-squared (*R*^2^) results for the two CVR models (with age, sex, group, and design as covariates; *indicates *p* < 0.0125). The first hypercapnia transition rate is in both models, while the first hypercapnia CVR measure is in Model-2.

Pearson correlation estimates between transition rate, CVR-1, CVR-2 and COPE-CVR (Figure 3B) demonstrate COPE-CVR is strongly correlated to the transition rate and non-parametric CVR estimates in Groups 1 and 3, but not in Group 2. Because this was not the primary outcome of this study, further analysis was not conducted.

## 4. Discussion

In this study we investigated BOLD-CVR responses in clinical samples and found the transition rate from hyper-to-normocapnia was significantly associated with the non-parametric CVR estimate of the subsequent challenge. The association between these two responses remained robust for all but one ROI, after refining the regression model to account for the first CVR response. These results demonstrated that the Sen’s Slope non-parametric approach was conducive for analyzing the intra-session effect of a CVR experiment. Simulated CVR responses demonstrated that the use of a non-parametric CVR dilation metric is comparable to the more conventional parametric method.

Simulated CVR responses supported the use of the Sen’s Slope as a viable alternative to a parametric CVR estimate, which is appealing when estimating individual CVR responses. The parametric CVR metric tended to show better performance (i.e., lower AIC) compared to the non-parametric estimate for fast CVR response scenarios. Meanwhile, the non-parametric estimation of BOLD response had a lower AIC value for the slower simulated responses. These simulation results are consistent with various CVR studies which reported delayed responses in cerebrovascular and white matter diseases populations (3, 9, 48–50). Parametric models consider additional covariates, such as hemodynamic response functions, to account for these delayed responses (51). In contrast, the non-parametric method does not rely on assumptions concerning the underlying BOLD signal, allowing the method to be used for different brain regions, populations, and/or experimental design. Assumptions of linearity between the BOLD-CVR response and the hypercapnia stimuli may be problematic in some circumstances (27). This assumption is circumvented by taking the delta of the CO_2_ signal. Shifting the PETCO_2_ time course, for example, can address discrepancies in CO_2_ arrival but may not account for faster and slower regional or between-patient responses. It has been demonstrated that the underlying sigmoidal relationship between vascular resistance and PaCO_2_ can often result in various non-linear responses to CO_2_, especially in diseased populations (28). By using a non-parametric approach, we can estimate BOLD signal change without having to assume how underlying physiology may affect the signal. Collectively, these simulation results again suggest that a non-parametric, model-free approach can be utilized to characterize a single vasodilatory event.

By separating temporal features of the BOLD-CVR experiment, we were able to assess the relationship between transition periods that occur during a hypercapnic challenge. The rise in BOLD signal during the administration of CO_2_-rich air can be used as an indirect measure of the physiological changes that occur due to CO_2_ inhalation, such as an increase in cerebral blood flow. The transition rate to normocapnia, which describes the rate at which the BOLD signal returns to baseline, provides an indirect measure of the physiological response to CO_2_ washout. We found evidence to suggest that the rate of BOLD signal decrease during a participant’s recovery from hypercapnia air has an influence on the BOLD signal’s rise induced by the following hypercapnia challenge. For CVR Model-1, the transition to normocapnia significantly influenced BOLD signal increase during the next challenge in all ROIs after accounting for age, sex, group, and experimental paradigm. This relationship revealed that a rapid recovery was linearly associated with a greater dilatory response during a subsequent administration of CO_2_ stimulus. CVR Model-2 further supported our primary finding by showing that the orthogonalized transition rate remained a significant feature to explain variance in the subsequent CVR estimate. Several explanations are worthy of discussion.

One reason for this observation may be that it is physiologically optimal for these transitions to and from hypercapnia to agree to avoid unbalanced and/or exaggerated hemodynamic responses. Furthermore, recovery of the basal CBF level may contribute to a carry-over influence on the subsequent hypercapnia BOLD response. We hypothesize that the physiological underpinnings of signal recovery influence factors related to vessel dilation, such as intracellular hydrogen concentration, baseline vascular tension and resistance (27, 52). The regulation of intracellular hydrogen has been hypothesized by Duffin et al. to be a key driver of the dilatory response to hypercapnia (53). This factor could potentially be influenced by the transition period to normocapnia prior to a subsequent hypercapnic challenge. For example, a short transition rate would clear PaCO_2_ quickly, minimizing the disturbance of PaCO_2_ on hydrogen concentration. This would allow for the vessel to return to its physiological baseline state and dilation to occur uninhibited by the effects of the previous challenge. In the case of a slower transition rate, the vessel may not return to baseline before the subsequent hypercapnia challenge occurs again. This would decrease vascular tension and resistance, increasing the baseline vascular diameter prior to dilation, and result in smaller fractional BOLD signal changes in response to CO_2_. While this phenomenon has not been observed in the cerebral vasculature, it has been studied in other vessels, such as the brachial artery. The relationship was observed in a conventional assessment of endothelial function by cuff-occlusion, which constricts blood vessels, before applying flow-mediated dilation. Aizawa et al. (54) and Harbin et al. (55) found evidence of an inverse relationship between vessel constriction and subsequent dilation during this assessment. These findings support the notion of common mechanisms across different vascular beds.

It is interesting to note that regional differences exist for these temporal associations as well. In both dilation models, the strongest association between the two periods occurred in the hippocampus. Hippocampal arteries are small in diameter and, depending on the vascularization pattern of the hippocampus, may have limited collateral supply for arterial blood compared to more vascularized grey matter regions. Thus, the lingering effects of vessel constriction and decreased capacity for vessels to dilate may be magnified. This implies that CVR features from within low-flow brain regions may be more dependent on the outflow of CO_2_. This may also explain why a region such as the centrum semiovale, a white matter region with limited arterial supply, also exhibits a strong relationship between temporal features of a CVR challenge. Conversely, the insula showed the lowest association between the two periods in CVR Model-1 and showed no association between the two features in CVR Model-2. The control of vascular resistance in the insula may be a factor due to a higher density of arterioles that can quickly react to local changes in arterial gas concentrations. The rapid ability to change vessel diameter suggests that the residual effects of hypercapnia (i.e., increased CBF) are less likely to impact a subsequent hypercapnia challenge. Also, the insula is in the middle cerebral artery territory, which is in physical proximity to the chemo-sensitive receptors of the carotid bulb. Hence, the insula may contain a higher density of arterial blood gas receptors that also contribute to quick reactivity to CO_2_. The regional differences observed in this study warrant further investigations into CVR regional heterogeneity.

We observed the influence of experimental design on dilation in the insula for CVR Model-2. This finding indicates that certain brain regions may be more sensitive to the delivery of CO_2_. Tancredi and Hoge (21) demonstrated that differences in the administration of CO_2_ can result in significantly different changes in cerebral blood flow. Our results reveal that this effect may be dependent on tissue type or vascularization. This observation suggests that careful considerations are needed if BOLD-CVR were to be pooled across sites, groups, and/or studies, if the CVR paradigms differ.

When briefly comparing the proposed non-parametric estimates and transition rate to a traditional CVR measure in the thalamus, we observed differences in the strength of correlation amongst clinical populations. For groups with cognitive concerns, the correlation between COPE-CVR and the newly derived measures (CVR-1, CVR-2, Transition Rate) is strong. Conversely, the sleep apnea group did not exhibit a similar pattern. These results suggest that traditional CVR measurements can benefit from alternative methods of measuring the BOLD signal response to gas manipulations.

There are limitations in this study that are discussed herein. While BOLD-CVR may be a suitable choice for this analysis, it does not provide a direct physiological measurement of vessel dilation or constriction. Therefore, the hemodynamic features explored in this study are only a proxy measure of the underlying physiological changes that occur during hypercapnia. Future work could investigate these features using a more direct measurement of vascular changes. We also recognize that CO_2_ variability was not captured as our primary focus was situated on the changes in BOLD signal. Thus, future work might include accounting for this variability and incorporating dynamic CO_2_ changes or further mitigation of CO_2_ variability through CO_2_ level stabilization during scans. More advanced BOLD imaging with multi-echo and high frame rate (simultaneous multi-slice) acquisition, cerebral blood flow ASL, or hybrid BOLD/ASL imaging would provide greater confidence that the CO_2_ challenges in succession are intricately linked. In the case of tissue segmentation for the different ROIs, the white matter region was manually segmented while other ROIs were segmented using automated software, which could confound CVR results. Furthermore, white matter hyperintensities were not removed from the centrum semiovale region nor was the ROI corrected for potential partial voluming effects that can occur during the transformation from T1-space to BOLD-space, which could affect the CVR response for this ROI (23). Future work would include optimizing the current masks to reduce potential influences of noise while also investigating additional regions of interest. Another limitation is the choice of non-parametric CVR estimation. As noted previously, the Sen’s slope is not without its limitations. Namely, fewer assumptions in this non-parametric method compared to parametric means that a noise term is not explicitly modeled. In this instance, future work may also include a comparison between linear models such as GLM as a typical method and our non-parametric model using Sen’s Slope for BOLD-CVR signal changes. It would also be intriguing to model the transition to normocapnia using a more sophisticated model to capture the non-linear recovery response.

A strength of this study was the opportunity to characterize CVR across clinical populations and experimental paradigms. This method attempts to address the ambiguity of BOLD-CVR data interpretation in diseased populations by providing an alternative method to characterize responses to hypercapnia. We did not observe group differences between the sleep apnea and cognitive impairment groups, indicating that these two clinical populations may either have similar physiological changes occurring in the brain or that the relationship between normo- and hypercapnia CVR physiological components (such as vasodilation and vasoconstriction) are preserved across different clinical populations. The impact of disease burden could be explored in future studies by assessing inter-group differences and including an age and sex-matched healthy control group.

This study attempted to develop an alternative method to quantify the dynamic BOLD-MRI responses to hypercapnia in clinical populations. We introduced a non-parametric measure of CVR and a transition to normocapnia rate. More work is needed to refine these measures, but our results indicate that the BOLD signal induced by a physiological response to hypercapnia can be influenced by a preceding hypercapnia challenge in various regions across the brain. Future work could compare how this response differs between healthy and non-healthy populations.

## Data availability statement

The raw data supporting the conclusions of this article will be made available by the authors, without undue reservation.

## Ethics statement

The studies involving human participants were reviewed and approved by Sunnybrook Health Sciences Centre Research Ethics Board. The patients/participants provided their written informed consent to participate in this study.

## Author contributions

K-JM-R contributed to study design, data analysis, interpretation, and writing. XJ, JP, RS, and AC contributed to data collection, interpretation, and writing. JR and DM contributed to study design, analysis, and edits. MT, JW, AL, SB, and BM contributed to study design, data collection, harmonization of study design and data collection, interpretation, and edits. All authors contributed to the article and approved the submitted version.

## Funding

This work was supported by funding from Fondation Leducq (16CVD05).

## Conflict of interest

DM contributed to the development of the RespirAct Gen3 breathing circuit (Thornhill Research, Toronto, Canada).

The remaining authors declare that the research was conducted in the absence of any commercial or financial relationships that could be construed as a potential conflict of interest.

## Publisher’s note

All claims expressed in this article are solely those of the authors and do not necessarily represent those of their affiliated organizations, or those of the publisher, the editors and the reviewers. Any product that may be evaluated in this article, or claim that may be made by its manufacturer, is not guaranteed or endorsed by the publisher.

## Footnotes

^1^ https://fsl.fmrib.ox.ac.uk/fsl/fslwiki/FEAT
